# Exploring public attitudes towards the new Faster Diagnosis Standard for cancer: a focus group study with the UK public

**DOI:** 10.3399/bjgp19X702677

**Published:** 2019-03-12

**Authors:** Marianne Piano, Georgia Black, Dorothee Amelung, Emily Power, Katriina L Whitaker

**Affiliations:** School of Health Sciences, University of Surrey, Guildford.; Department of Applied Health Research, University College London, London.; School of Health Sciences, University of Surrey, Guildford.; Cancer Intelligence Team, Cancer Research UK, London.; School of Health Sciences, University of Surrey, Guildford.

**Keywords:** cancer, early diagnosis, Faster Diagnosis Standard, general practice, patient experience, referral and consultation

## Abstract

**Background:**

The Faster Diagnosis Standard (FDS) is to be introduced in England in 2020. This standard is a new policy in which patients should have cancer ruled out or diagnosed within 28 days of referral.

**Aim:**

To explore public attitudes towards the FDS within the context of their recent referral experiences.

**Design and setting:**

Four 90-minute focus groups (two in Guildford, two in Bradford).

**Method:**

Participants aged >50 years without a current cancer diagnosis (*N* = 29), who had completed certain diagnostic tests, for example, ultrasound, and received results within the last 6 months were recruited. Age, education, and sex were evenly distributed across groups through purposive sampling.

**Results:**

The largest cause of concern was the waiting process for obtaining test results. Most had experienced swift referral, and it was difficult for participants to understand how the new standard could impact upon time progressing through the system. Responsibility for meeting the standard was also a concern: participants did not see their own behaviours as a form of involvement. The GP’s role was conceptualised by patients as communicating about their referral, establishing patients’ preferences for information, and continued involvement at each stage of the referral process. The standard legitimised chasing for test results, but 28 days was considered too long.

**Conclusion:**

Patients should be asked what they would like to know about their referral. GPs should be more transparent about the referral process and the potential for a lack of clarity around next steps.

## INTRODUCTION

The diagnostic process for cancer is complex and multifaceted,^1^ with ongoing public pressure to expedite and improve it. The NHS Five Year Forward View included a recommendation to develop and implement the Faster Diagnosis Standard (FDS), in which patients will receive a diagnosis or all-clear for cancer within 28 days of referral for diagnostic testing.^2^ This new standard aims to facilitate a patient-centred, flexible, and rapid approach to cancer diagnosis/non-diagnosis. It is different from existing standards such as urgent referrals, that is, where patients are seen by a specialist within 2 weeks,^3^ because it extends further along the cancer care pathway to include the time it takes to confirm or rule out a diagnosis, rather than simply time to the first specialist appointment. It is hoped that implementing the standard will expedite cancer diagnosis, and provide faster reassurance for the majority of people who will not have a cancer diagnosis.

Evidence surrounding missed opportunities in cancer diagnosis emphasises the importance of patient empowerment to minimise risk of prolonged diagnostic intervals. Patients need to be engaged, or invested, to follow up on test results, re-consult their GP if symptoms persist or worsen, and attend follow-up appointments.^1^ Although patient preferences have been explored regarding communication of a confirmed cancer diagnosis,^4^^–^6 and whether or not to initiate referral,^7^^,^^8^ there has been limited study of patient communication preferences during referral for diagnostic testing to confirm or rule out cancer.^9^ Existing studies have used vignette methodology to quantitatively explore patient preferences for referral, for example, demonstrating preferences for investigation at 1% cancer risk,^7^^,^^8^ whereas previous qualitative research with people referred on an urgent referral pathway focused on symptom appraisal and help seeking, rather than exploring referral experiences.^10^

To date, patient views at the point of referral for diagnostic tests to possibly confirm, or rule out, cancer have not been explored and there has been no research to explore public attitudes towards the new FDS. As such, little guidance exists for primary care staff regarding navigation of these conversations at point of referral. There may also be reticence in raising the possibility of cancer at this stage of the diagnostic process, when the majority (90%) of people referred on cancer pathways have cancer ruled out.^3^ This study explored public attitudes towards the FDS, within the context of people’s recent referral experiences.

## METHOD

### Participant selection

The authors conducted focus groups in two different geographical areas (Guildford, Southeast England; Bradford, Yorkshire and Humber) to access a wide range of opinions and referral experiences, and enable participant reflection and discussion regarding the FDS. Guildford was chosen as the study’s southern site for practical reasons as it is where the study team is based. The authors chose Bradford to include an area with a different demographic profile. Bradford has a larger population (approximately 500 000 versus 140 000) and is more ethnically diverse (64% versus 84% of people identify as white British respectively).^11^^,^^12^ Neither site was involved in the pilot programme for the FDS.

**How this fits in**

The Faster Diagnosis Standard (FDS) aims to ensure patients will have cancer ruled out or diagnosed within 28 days of referral for diagnostic testing. There is currently no evidence demonstrating how the public may view this standard. This study highlights the pivotal role of the GP within the FDS, as perceived by the public. Recommendations are made to facilitate GP–patient conversations at the point of referral under the FDS.

Focus groups allow examination of people’s health service experiences, enabling group discussions to explore attitudes in a way that may be less accessible with individual interviews.^13^ Eligible participants were approached by market research agency Saros Research Ltd through their established volunteer databases over a 4-week period. Purposive sampling ensured that location (rural or urban), sex, and education level were evenly distributed among the sample. The authors chose to run four focus groups following a recent review suggesting that 90% of themes are discoverable within three to six focus groups.^14^ Sample size (*n* = 6–8 per focus group) was determined based on recommended numbers for focus groups.^15^

To be eligible, participants had to:

- be aged ≥50 years; and
- have been referred from primary care for one or more of the following diagnostic investigations within the last 6 months: ultrasound scan, computed tomography/magnetic resonance imaging (CT/MRI) scan, mammogram, chest X-ray, colonoscopy/sigmoidoscopy, endoscopy (other), biopsy. These investigations were taken from the authors’ previous research exploring the impact of an all-clear diagnosis on subsequent help-seeking for potential cancer symptoms.^16^

The majority of referrals under the FDS are likely to have cancer ruled out,^3^ thus excluding people with a previous or current diagnosis of cancer ensured a homogeneous group to focus on referral experiences and attitudes towards the FDS.

Two focus groups were conducted in each location (Guildford: University of Surrey; Bradford: Community Empowerment Network (CNet) community centre, Manningham). Participants provided informed consent and completed a demographic questionnaire before taking part. Focus groups lasted approximately 90 minutes.

### Focus groups

The topic guide was developed with input from cancer survivors, members of the public, and academics external to the research team (the topic guide is available from the authors). Participants were invited to share the most important or meaningful thing to them when being referred by their GP for a clinical test. These topics were then explored more fully, with participants asked to reflect on how their referral experience could have been improved. Following these initial reflections, a definition of the FDS was presented to the group:

*‘The Faster Diagnosis Standard is a new care standard, so people referred by their doctor for certain clinical tests will find out whether or not they have cancer within 28 days of the referral.’* 1

Participants were asked for their thoughts on the standard, and in what ways their experience of being referred, tested, and receiving the results might have been influenced, had they been referred under the standard. Participants were also asked whether they thought they had a role to play in meeting the standard, and to explain their reasoning.

### Analysis

Focus group discussions were digitally audio-recorded and transcribed verbatim. Transcripts were deductively analysed using thematic analysis.^17^^,^^18^ For each focus group a single researcher familiarised themselves with the transcript, abstracting the conversation for analysis meetings with three others from the research team. The initial setting of themes within a framework was reviewed for fit, and further refined by the research team. Refinement was inductive and derived from concepts grounded in the data using mind maps. This allowed concept integration into meaningful themes, and associations between themes to be explored. Key recommendations were generated and conveyed at an event at Cancer Research UK attended by academics external to the project, Cancer Research UK staff, and patient representatives. Event feedback was used to finalise the analysis and recommendations.

## RESULTS

### Participants

The focus groups had a total of 29 participants and a balanced sex distribution (males *n* = 14, females *n* = 15) and settlement area (urban *n* = 13, rural *n* = 16), and included a range of education levels (Table 1). Many experienced more than one clinical test (*n* = 16). The most commonly reported clinical tests were ultrasound (59%, *n* = 17) and CT scan (38%, *n* = 11). Less common tests included mammogram (7%, *n* = 2) and biopsy (3%, *n* = 1). Participants reported a range of comorbidities (Table 2).

**Table 1.** Demographic characteristics, *N* = 29

**Characteristic**	***n***
**Age**	
Mean (SD), years	58.76 (6.39)

**Sex ratio**	
Male: female	14:15

**Ethnicity**	
White/white British/white other	25
Asian/Asian British	3
Black/African/Caribbean/black British	1

**Location**	
Southeast (Guildford)	15
Yorkshire/Humber (Bradford)	14

**Environment**	
Urban	13
Rural	16

**Education level**	
Degree or higher degree	15
Higher education qualification below degree level	3
A-levels (AS levels/Advanced diploma/equivalent)	3
O Level/GCSE	8

**Marital status**	
Single/never married	2
Married/living with partner	23
Divorced/separated	3
Widowed	1

**Car ownership**	
No	2
Yes, one	12
Yes, two or more	15

**Living arrangements**	
Home owned outright	10
Home owned with mortgage	16
Renting from local authority or housing association	2
Renting privately	1

**Employment status**	
Employed full-time	3
Employed part-time	10
Unemployed	1
Self-employed	5
Full-time homemaker	2
Retired	7
Disabled or too ill to work	1

**Smoking status**	
No, has never smoked	17
Not now, but used to smoke	9
Yes, is current smoker	2
Missing	1

**Test received**	
Ultrasound	17
CT scan	11
Endoscopy (other)	4
Mammogram	2
Chest X-ray	6
Colonoscopy/sigmoidoscopy	6
Biopsy	1
PSA	3

**Presence of disease in close family/extended family/friend**	
Heart disease	18
Cancer	24
Asthma	11

*CT* = *computed tomography. PSA* = *prostate-specific antigen.*

**Table 2.** Comorbidities experienced by participants, *N* = 29

**Condition**	***n* (%)**
Arthritis	10 (34.5)
High cholesterol	5 (17.2)
Hypertension	6 (20.7)
Depression	4 (13.8)
Circulatory problems	4 (13.8)
Diabetes	3 (10.3)
Stroke	1 (3.4)
Chest problems	4 (13.8)
Heart problems	2 (6.9)
Visual impairment	1 (3.4)
Thyroid problems	2 (6.9)
Bowel problems	1 (3.4)
Swallowing problems	1 (3.4)

### Cynicism about how the FDS will improve existing referral speeds

The FDS aims to standardise referral speed and diagnostic pathways, yet participants described a referral system already felt to be speedy in terms of reaching the point of diagnostic testing:

‘I am partly worried about the failing NHS and I thought I’m just going to get fobbed off here and told to go take a pill, and so I was quite impressed that I got access to all these specialist services, and as quickly as I did.’

(Bradford [B]1, male [M])

In this context, the FDS was not considered fast enough. Some expressed concerns that FDS roll-out may ultimately extend waiting times, running counter to its intended effect:

‘If that’s the new standard, 28 days, then we’ve got to be frightened of what we’ve got now, you know, that’s the frightening thing.’

(B2, M)

*‘So what happens is that as soon as 28 days appears anywhere that becomes the standard, rather than the last resort, so when suddenly you go, well we’ve got 28 days, we’ll give them a … we’ll get in touch with them in 3 weeks’ time* […].*’*

(G [Guildford] 2, M)

This reticence was also apparent when groups discussed existing speed-based ‘targets’. Awareness about what these targets were or how the new standard might fit was limited:

*‘There’s something about this already, so certain types of cancers they could, within 6 weeks if you go to a, I think breast cancer is one of them, the doctors, so that’s only a couple of days, at the moment it’s 6 weeks they tell you that when you go to the doctors for certain types of cancers, right, you will be on the path, so if you’re looking at 28 days, so now we’re talking working days, that’s only 2 days shorter and that’s available at the moment, so …* ’

(G1, M)

### Reassurance or support valued as highly as speed

Other aspects of GP interactions arose that were felt to be as important as speed. For example, the GP was perceived to have a pivotal role in providing information and reassurance regarding the referral, based on their knowledge of the patient’s information needs, which were considered highly individual:

*‘It depends on what the problem is and of course the individual, so the GP actually knows the individual, they can actually judge whether they need a lot more reassurance and information, as far as I was concerned I wanted information and, you know, being given options, and it’s pretty straightforward, but I know that some people would actually want a lot more reassurance, so I think it is key to it … GP actually knowing the patients* …*’*

(B2, M)

‘There are some of us that want to know everything by the time we leave the GPs and others would prefer to be in, you know, ignorance, so it must be very difficult for them to know what to do.’

(G2, M)

People also emphasised the importance of feeling listened to and validated by the GP’s response and decision to refer:

‘Being listened to I think, so it’s being heard, and my GP was fine, has really really been … it was really quick, it couldn’t have been quicker, but it was feeling … I suppose it’s being listened to and then almost like being believed.’

(G1, female [F])

### Knowing what to expect through more transparent referral processes

Participants described a lack of transparency in the referral process, and concern about getting lost in the system. This feeling was exacerbated by not knowing what to expect, and/or being unable to draw upon past experiences due to inconsistency between one referral and the next:

*‘*… *and then right OK, there’s an appointment I come, I don’t actually know which hospital it is, I presume it’s the local one so until I get the letter I actually haven’t got that confirmation, but I’d just like them to get on with it and, but obviously I have to know exactly where I’m going, that would my only thing, I would like to know exactly where I’m going on the day and I know I’m going to be seen and not passed from pillar to post.*’

(G1, F)

*‘Yeah, I think that’s probably it, yeah, the disparity between everyone’s experiences, and also within your own experiences, of similar situations occurring, and suddenly things happen, and it is that lack of consistency that I think frustrates people so much.*’

(G2, M)

Participants described themselves as passive actors in a system that was ‘done to them’ or in which ‘suddenly things happen’, suggesting that individuals experience a loss of agency when entering the referral system, and feel pulled along by various processes. Increasing consistency and transparency of these processes was perceived as a means to regain this sense of agency:

*‘Sometimes it’s also about feeling like you have some control of what’s going on, rather than it’s being done to, so maybe trying to get more of a … improve the standard of that communication and be more consistent, so wherever you are and wherever, you know, whatever part you’re in, it’s a standard way of doing things.*’

(B2, F)

Opaque and inconsistent processes were exemplified within participant experiences of obtaining test results, having to chase for them or receiving them by letter without support:

*‘You’re working, and you’ve sort of trying to find time in-between your work to make phone calls and then you can’t get through or they say they haven’t got the results and then my surgery did tell me to ring the hospital, where I thought really they should have got the results rather than me having to ring up, and then I had quite a negative experience with the medical secretary and I sort of said to her, “Well what if you’d got cancer then, what if it’s something serious?” and she just brushed it off and she just said, “Well you have to wait,” … I didn’t have a very positive experience with the results process.*’

(B1, F)

‘… *certainly, when I was waiting for my results, I’d asked the consultant anyway and he’d give me a pretty good idea, and then I went to wait, and that is quite, that’s awful because it’s just like a letter just drops and they don’t even bother ringing up* […]’

(B1, F)

It was suggested that the FDS could help by setting expectations and ensuring accountability:

*‘The good thing about that sort of measurement though is it is from the patient’s point of view, you will get your result within 28 days, you know, that you can understand that and there’s a lot of detailed questions about it, but it’s aimed at telling you what to expect.*’

(G1, M)

### Clarifying the role of patients in the referral process

Participants wanted to be offered the choice of where and when to attend follow-up appointments, to be seen as quickly as possible, and know where they were in the system. Systems enabling this kind of independent involvement, for example, ‘choose and book’, were perceived as beneficial to re-establish a sense of agency, based on direct experience:

*‘The thing that swung it for me is that I deliberately chose to go to the one that was further away so it was longer to drive to, but I would be seen quicker and immediately without waiting in the waiting room area, and it was, it was instant, I was just straight in, straight out, and a free car park!* ’

(G1, F)

‘I know I’m not lost in the system because I am making the appointment and I’m going to get an answer today and I’m going to get something in my diary today because I’m the one doing it and I haven’t got to wait at home for the referral.’

(G2, F)

However, participants did not always consider their involvement legitimate, perceiving common strategies such as taking cancellations to get an earlier appointment as manipulating the system:

*‘I just rang direct, and said, “Look, have you got any cancellations?” and I got one the next day* … *there’s a lot of people that cancel, and don’t turn up, and they have spaces and they know if they can fit you in for that amount of time because they know what you’re going for, it’s easy done. I know the system.’*

(B2, F)

GPs were considered facilitators in this process because they had access to information that patients might not, and could also legitimise these ways of navigating the referral process:

‘I think a discussion with GP would be an important thing here, because GPs are in, they have the authority to direct you to the certain location for which consultant, which hospital you should go for this test, that hospital, the GPs would know all this, that hospital takes longer and getting the results back, so it would be up to the GP to sort of see what the patient wants …’

(B1, M)

When people considered their role in meeting standards such as the FDS, it was felt that patients should not need to be involved as the responsibility lay with health professionals:

*‘The point of having performance standards such as this is to drive up standards generally on the performance of the cancer team or whatever, you know, and to minimise the requirement for us the users to be being involved in that process, the responsibility for performance lies with the leadership team, or within the NHS, or wherever it is, and therefore in my view, you know, there shouldn’t be a need for the users to have to get involved in this because the whole point of having those performance standards is to make sure that the service that’s being delivered is efficient and people know generally within 28 days.*’

(B2, M)

Patients were considered to be passive players in a complex system, as described previously. However, some recognised that the public could play a role in the process and meeting the 28-day standard:

*‘If everybody has a role then the system should become more seamless anyway, if everybody takes ownership for their own condition or responsibility of the appointments and things, like turning up and not cancelling it at the last minute and having that role, then surely that would make it easier for them to stick to the 28 days.*’

(G1, F)

### FDS considered complex to measure or deliver

Cynicism was widespread regarding achievement of FDS targets in primary and secondary care, within the constraints of a compartmentalised system:

*‘It doesn’t rely on one person, or one department, there’s so many variables, if you went into a department and they said, “We will do this and get you in the following day,” you would say, “Oh OK,” you know, we would get on board with that, but going from the GP to the consultant to the radiologist to somebody else, to somebody else, there’s so many variables in the process I don’t think it can be achieved.*’

(B1, M)

An even more extreme view was that it represented a mere ‘tick box’ exercise, lacking meaningful change for patients:

*‘You can tell that amongst the group we look at that and we’re immediately thinking, how are they going to wriggle and fiddle to meet that in a way that we will then think, well you met the target, but it’s still rubbish, you know, that there’s too much missing, there’s too much ambiguity in that and you don’t measure one thing, you know.*’

(G1, M)

Focus group conversations arose from a general understanding that the standard was designed to expedite cancer diagnosis. However, when the ‘ruling-out’ angle was considered, some were doubtful that this would provide answers, or a relevant diagnosis within the specified timeframe:

*‘You go for a test and it’s usually to rule out something, that’s what you get told isn’t it, “We want to just rule a few things out,” so you have whatever the test is, and it does rule it out, but then that’s the end of the line, it stops, and that’s what I find really annoying, I’ve still got the symptoms, I’ve still got the pain, I’ve still got what was the reason I came to see you in the first place*.*’*

(G1, F)

‘It says certain clinical tests, so who decides what tests are going to be done, because I mean are we saying that some tests will discover all cancers, or we think you might have this cancer therefore we’re going to give you this test, if it comes back clear you don’t have any cancer, it’s a bit ambiguous.’

(G2, M)

## DISCUSSION

### Summary

The researchers in the present study explored public attitudes towards being referred by a GP for clinical tests and, for the first time, used this backdrop to explore attitudes towards the new Faster Diagnosis Standard, which will be introduced in England in 2020. Males and females referred by their GP for diagnostic testing in the past 6 months discussed their views and experiences in four focus groups. Most had experienced fast referral, and found it difficult to understand how the new standard could decrease time spent progressing through the system. Speed was just one factor that was considered important when being referred for clinical tests: reassurance, feeling listened to, and transparency within the process were also highly valued. Where responsibility lay for meeting the standard was considered unclear, and participants did not necessarily view their own behaviours to secure an appointment or obtain test results as a form of involvement in this. The GP’s role was conceptualised as communicating with them about their referral, establishing their information needs, and maintaining involvement at each stage of the referral process. Reflecting on their own recent experiences, one of the main concerns was the process of waiting for and obtaining test results: people felt strongly that they did not want to receive results by letter and should not have to chase them.

### Strengths and limitations

These findings are the first to illuminate public attitudes towards the FDS. By drawing upon recent referral experiences of the public, this study provides insight into factors for consideration by doctors and wider stakeholder groups, ensuring public views are taken into account as the standard is rolled out. A major strength is the development of key recommendations in collaboration with patients with cancer, members of the public, and academic experts in primary care and cancer diagnosis. It should be recognised that these findings are specific to those who were given an all clear for cancer, but this comprises 90% of people referred for clinical tests in this context.^3^ However, this means the authors cannot draw conclusions about the experiences or attitudes towards the FDS of those with a previous or current cancer diagnosis.

The authors included clinical tests from their previous research in primary care^16^ but acknowledge that the broad spectrum of tests meant not everyone in the present study would have been referred according to a cancer pathway and there would have been variation in how people received their test results. However, the authors felt it was important to keep the range broad because, first, their main aim was to use people’s referral experiences to anchor the conversation about the FDS and, second, that people may not have known that they were referred on a cancer pathway and so may have been unnecessarily excluded. Future research could further delineate patient experiences of referral by exploring different ways of accessing diagnostic tests, for example, ‘straight to test’, referral to a specialist or multi-diagnostic centre and so on, and how these influence the patient pathway.

Importantly, views of GPs and other healthcare professionals should be considered before FDS roll-out, as recent evidence suggests GPs in the UK were less likely to retain responsibility for patient follow-up compared with non-UK GPs.^19^ Finally, purposive sampling helped promote diversity, with equal sex representation and inclusion of differing ethnic groups, educational backgrounds, and geography, though the authors acknowledge that another limitation is that the sample was still mainly white British and well educated.

### Comparison with existing literature

A recent King’s Fund-commissioned inquiry paper^20^ highlighted the importance of a feeling that progress is being made through the system following GP referral,^21^ suggesting that providing information regarding the referral process can increase patient satisfaction.^22^ This supports the need for balance between speed of referral or testing and care aspects such as information, trust, and communication.

In the present study, the GP was identified as having a key role in maintaining this balance, including signposting to appropriate services and determining how much information to provide about the referral, based on their knowledge of the patient and their support needs. Previous studies also identified the importance of patients feeling they are listened to, or taken seriously, by the GP,^23^^,^^24^ receiving advice from the GP regarding choice of hospital,^25^ and having their information and support needs understood. In addition, it was also considered important for GPs to have time to provide information and reassurance.^26^^,^^27^

Difficulties obtaining test results are a common experience for patients.^28^^–^30 In the present study, participants sometimes obtained results from the specialist but in the majority of cases obtained them from the GP, or had to wait for a letter. Letters have been identified previously as a slow form of communication for results of importance,^28^^,^^31^^,^^32^ and communication by letter held strong negative connotations for participants in the current study. Similar to findings by Litchfield *et al*,^30^ patients felt that while it is necessary to chase up results when they had not been communicated, it should not have to fall to them to do so.

Participants saw the FDS as a way of clarifying when they could rightfully phone to chase test results, but the majority felt 28 days was too long to wait for suspected cancer, and they would contact providers sooner than this. Previous qualitative studies have shown that patients who went on to have a cancer diagnosis considered a 2-week wait to be too long, provoking anxiety.^23^^,^^33^ Awareness of current standards for diagnostic referral for cancer was low within the group, with both over- and underestimates of waiting timeframes reported by participants within discussions. This finding is supported by other qualitative research indicating low awareness of current urgent referral pathways for cancer diagnosis.^34^ While participants in the present study did not have a diagnosis of cancer, many conveyed an expectation that their progress through the pathway would be speedier if cancer was highly suspected. Low awareness of current norms may therefore contribute to the perception that 28 days is too long to wait to have cancer ruled in or out.

### Implications for practice

The study findings provide an evidence base upon which to make recommendations that may help GPs and other healthcare professionals when the FDS is introduced. These recommendations are summarised as follows:

- Patients should be asked what they would like to know about the diagnostic testing process, encompassing referral, specialist input, testing, and obtaining results.
- Where appropriate, GPs should be more transparent about the referral process and the potential for lack of clarity around next steps, timescales, and outcomes.
- Patients should know that it is acceptable to make use of opportunities perceived as ‘manipulating the system’, for example, phoning up to offer to take a cancellation at short notice. However, care needs to be taken to avoid adding to symptom burden by forcing responsibilities upon the patient.
- Results should be communicated according to the patient’s preference, and ideally not via letter (without other support or follow-up).

Among people recently referred for clinical tests by their GP, speed of referral was merely one of a number of priorities for improving their experience. The introduction of the FDS may therefore provide an opportunity to address these other priorities, not only by providing guidance to GPs who face dynamic and complex diagnostic processes, but to empower the public to play a more central part.
